# Supplementary material for: Modeling dry eye with an air–liquid interface in corneal epithelium-on-a-chip
Source: Sci Rep. 2024 Feb 20;14:4185. doi: 10.1038/s41598-024-54736-z (PMC10879145; doi:10.1038/s41598-024-54736-z)
Supplement: Supplementary file 2 — Supplementary Information 2. [file 41598_2024_54736_MOESM2_ESM.docx]

Supplementary Information

**Modeling dry eye with an air-liquid interface in corneal epithelium-on-a-chip**

**Rodi Kado Abdalkader^1^***^†^**, Romanas Chaleckis^2,3^, Takuya Fujita^1,4^, and Ken-ichiro Kamei ^5,6,7^**

^1^ Ritsumeikan Global Innovation Research Organization (R-GIRO), Ritsumeikan University, Shiga, Japan

^2^ Gunma University Initiative for Advanced Research (GIAR), Gunma University, Maebashi, Japan

^3^ Department of Occupational and Environmental Health, Nagoya City University Graduate School of Medical Sciences, Nagoya, Aichi, Japan

^4^ Department of Pharmaceutical Sciences, Ritsumeikan University, Shiga, Japan

^5^ Institute for Integrated Cell-Material Sciences (WPI-iCeMS), Kyoto University, Kyoto, 606-8501, Japan

^6^ Programs of Biology and Bioengineering, Divisions of Science and Engineering, New York University Abu Dhabi, Abu Dhabi, United Arab Emirates

^7^ Department of Biomedical Engineering, Tandon School of Engineering, New York University, Brooklyn, NY 11201, USA

*Corresponding author.

*E-mail address:* rodi@fc.ritsumei.ac.jp

^†^Earlier known as Rodi Abdalkader


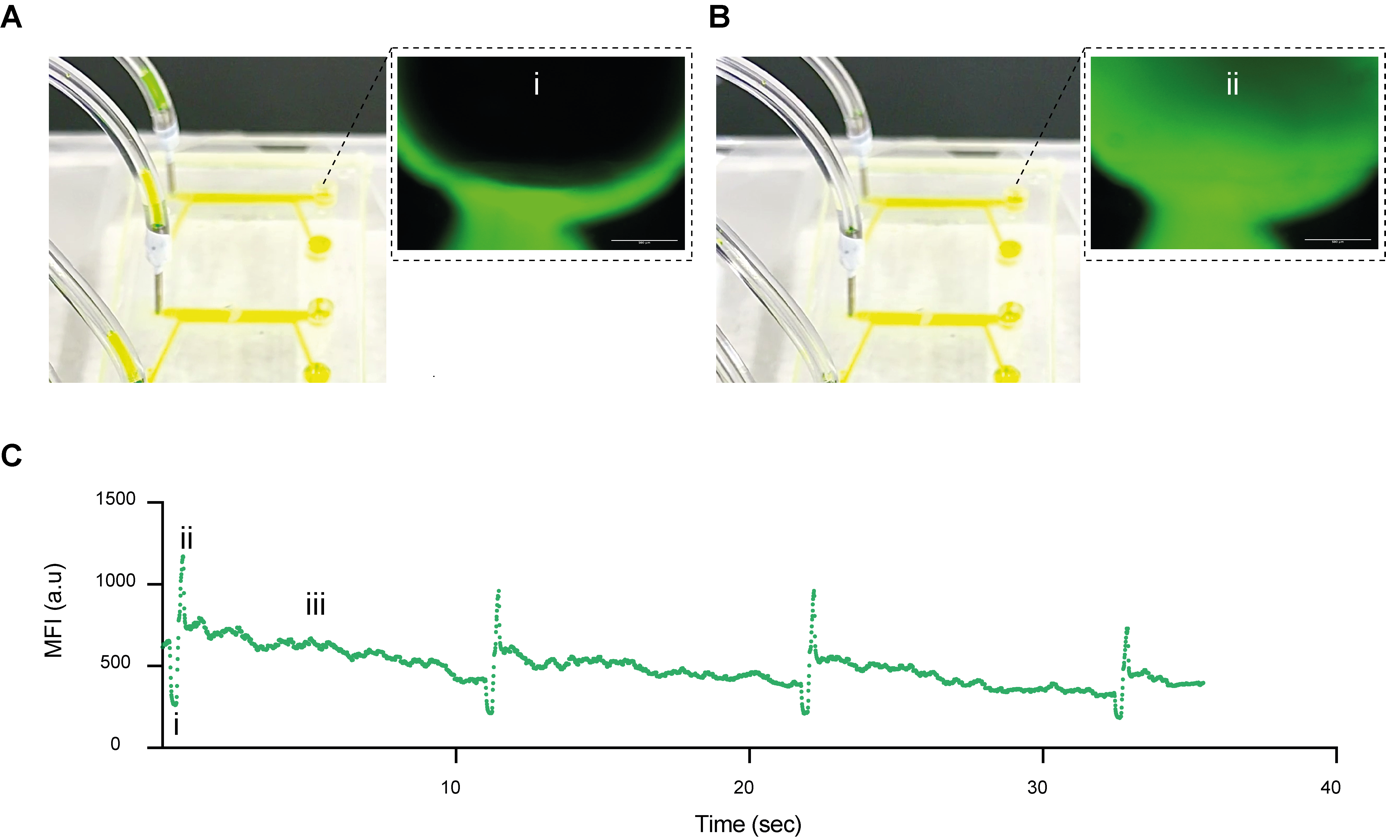


**Supplementary Figure 1. The process of AL stimulus under cell-free conditions.** (A) Air withdrawal (i) (A) Air infusion (ii). The green signal is captured from the sodium fluorescein solution. (C) Graph that showcase the mean fluorescence signal in the reservoir area during air phase (i), liquid phase (ii), and pause phase (iii).

**
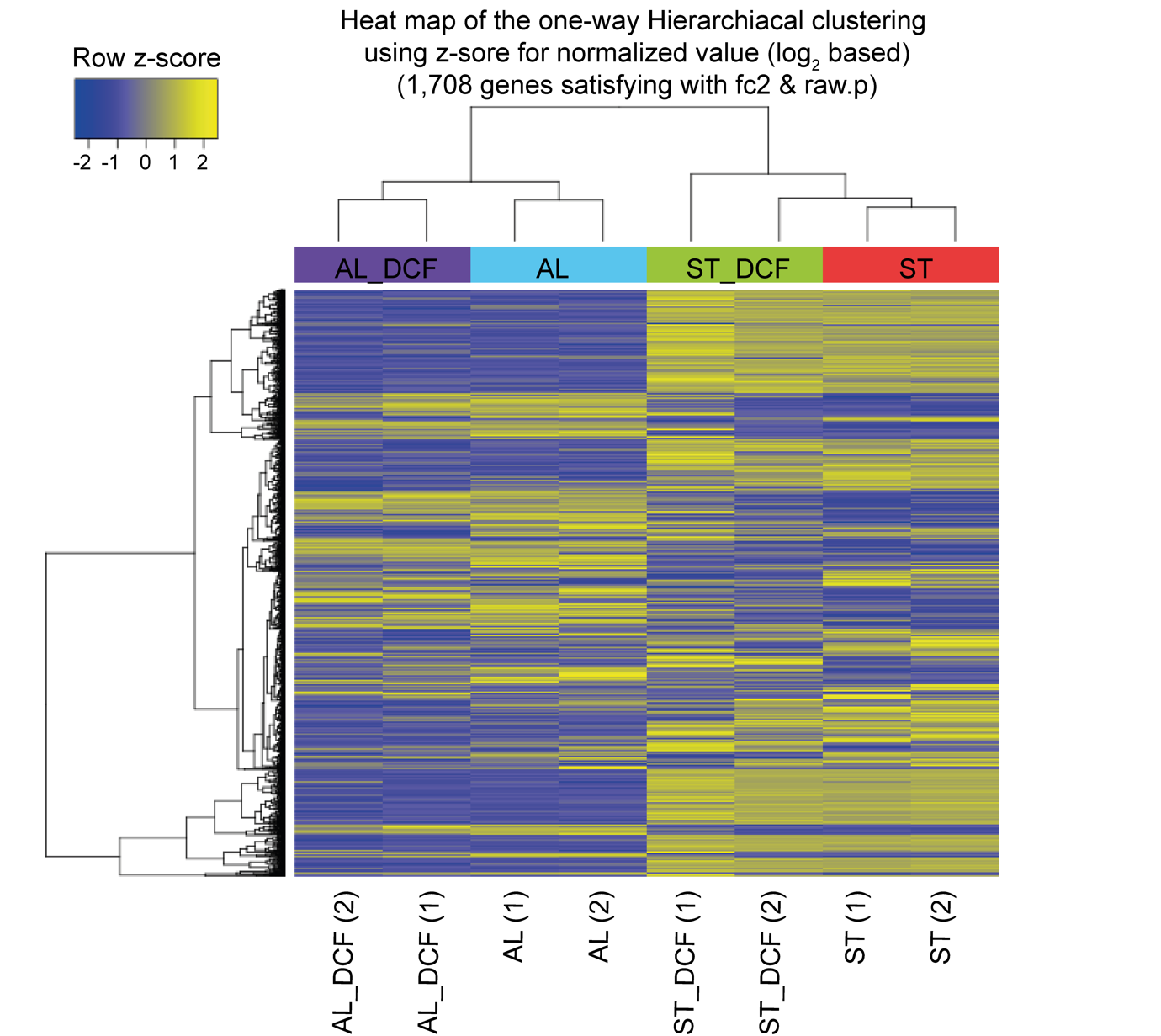
**

**Supplementary Figure 2.** Heatmap of the one-way Hierarchical clustering using z-score for normalized values (Genes with 2-fold change and *p*-value <0.05)


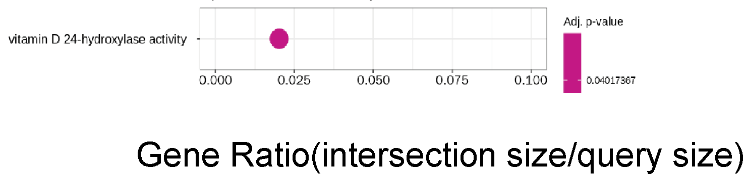


**Supplementary Figure 3.** GO pathways of DEGs in of ST_DCF vs ST. Genes with 2-fold change and *p*-value <0.05 was applied.

**
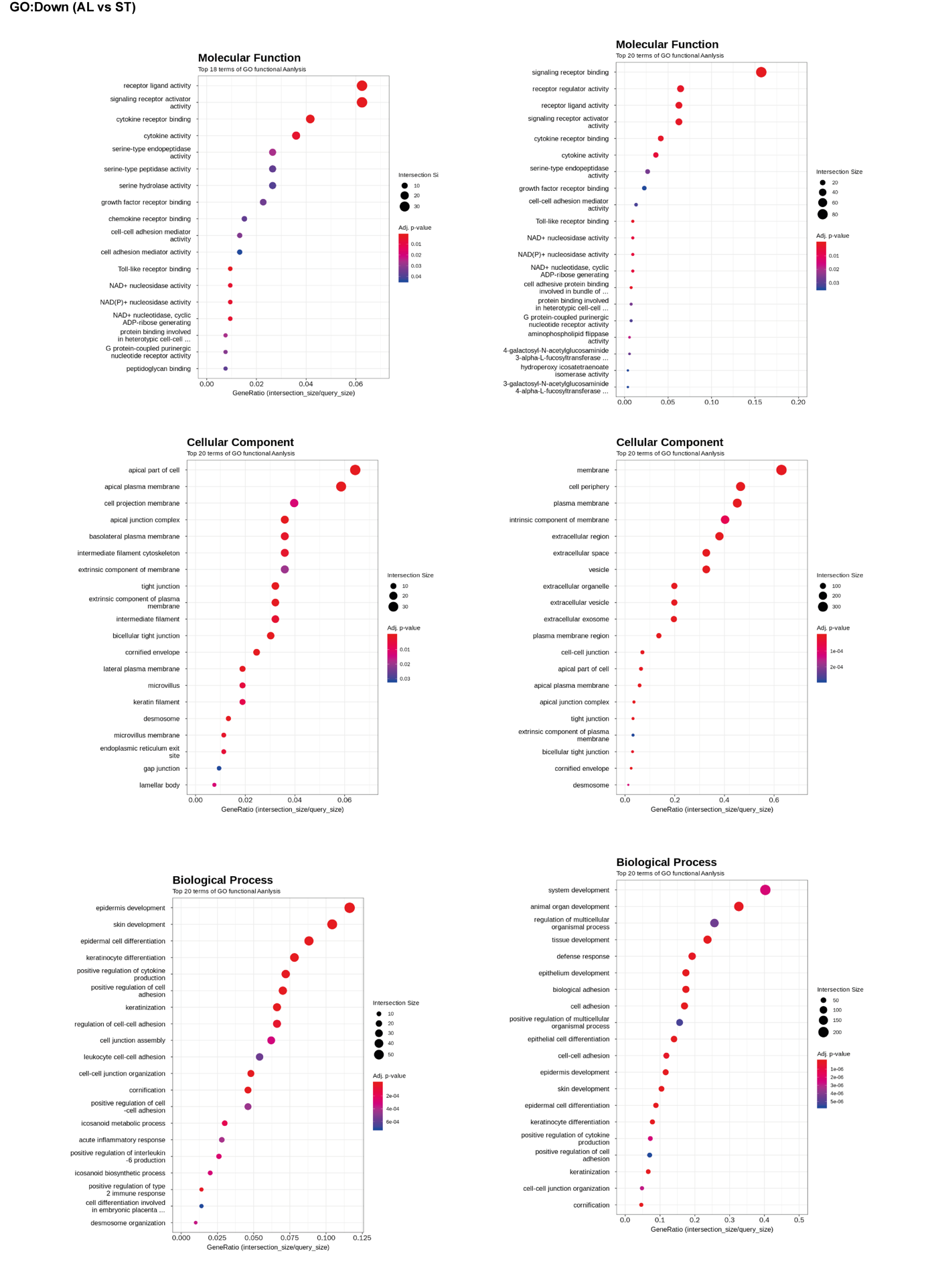
**

**Supplementary Figure 4.** GO pathways of DEGs in of AL vs ST. Genes with 2-fold change and *p*-value <0.05 was applied.


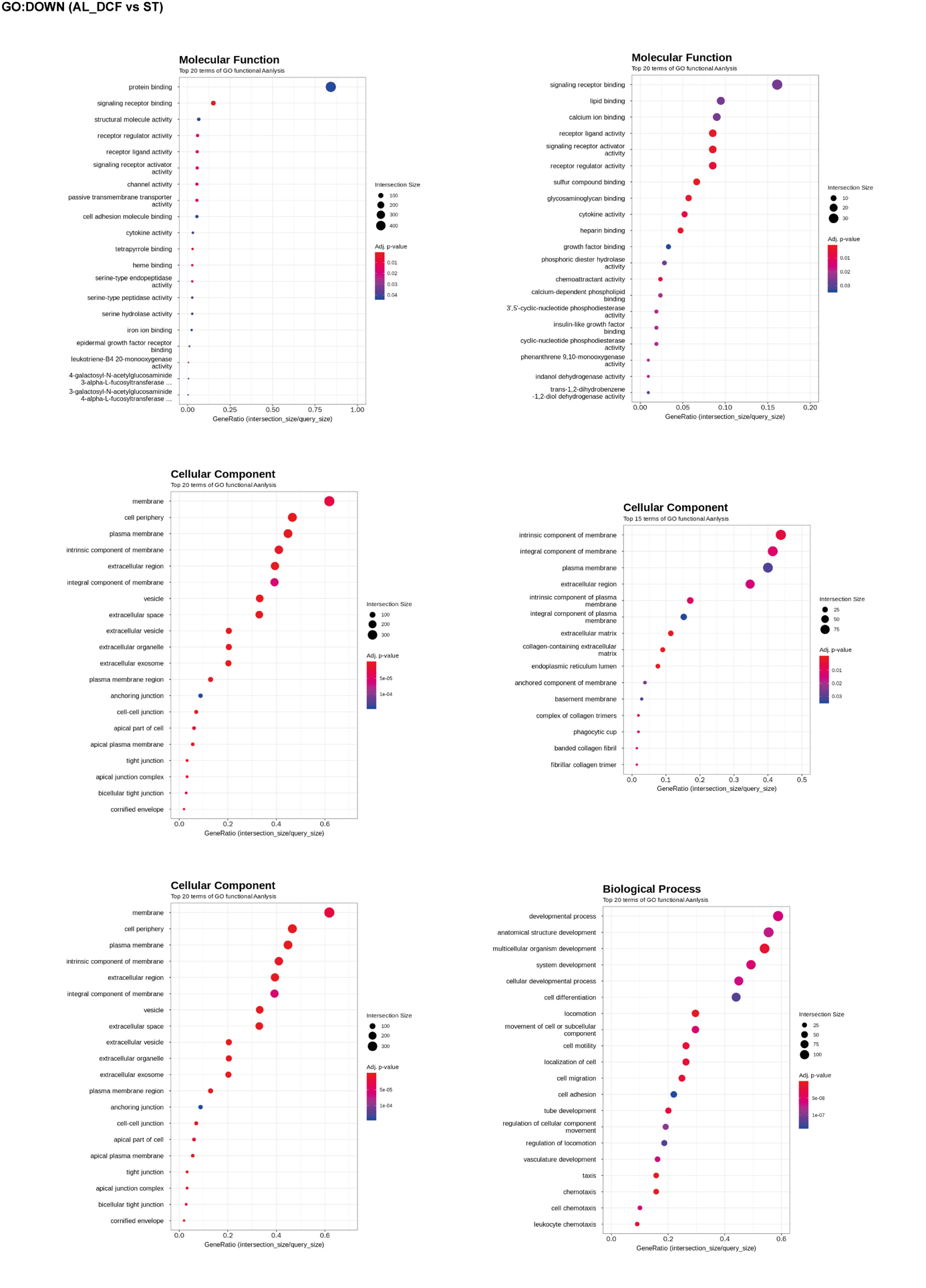


**Supplementary Figure 5.** GO pathways of DEGs in of AL_DCF vs ST. Genes with 2-fold change and *p*-value <0.05 was applied.


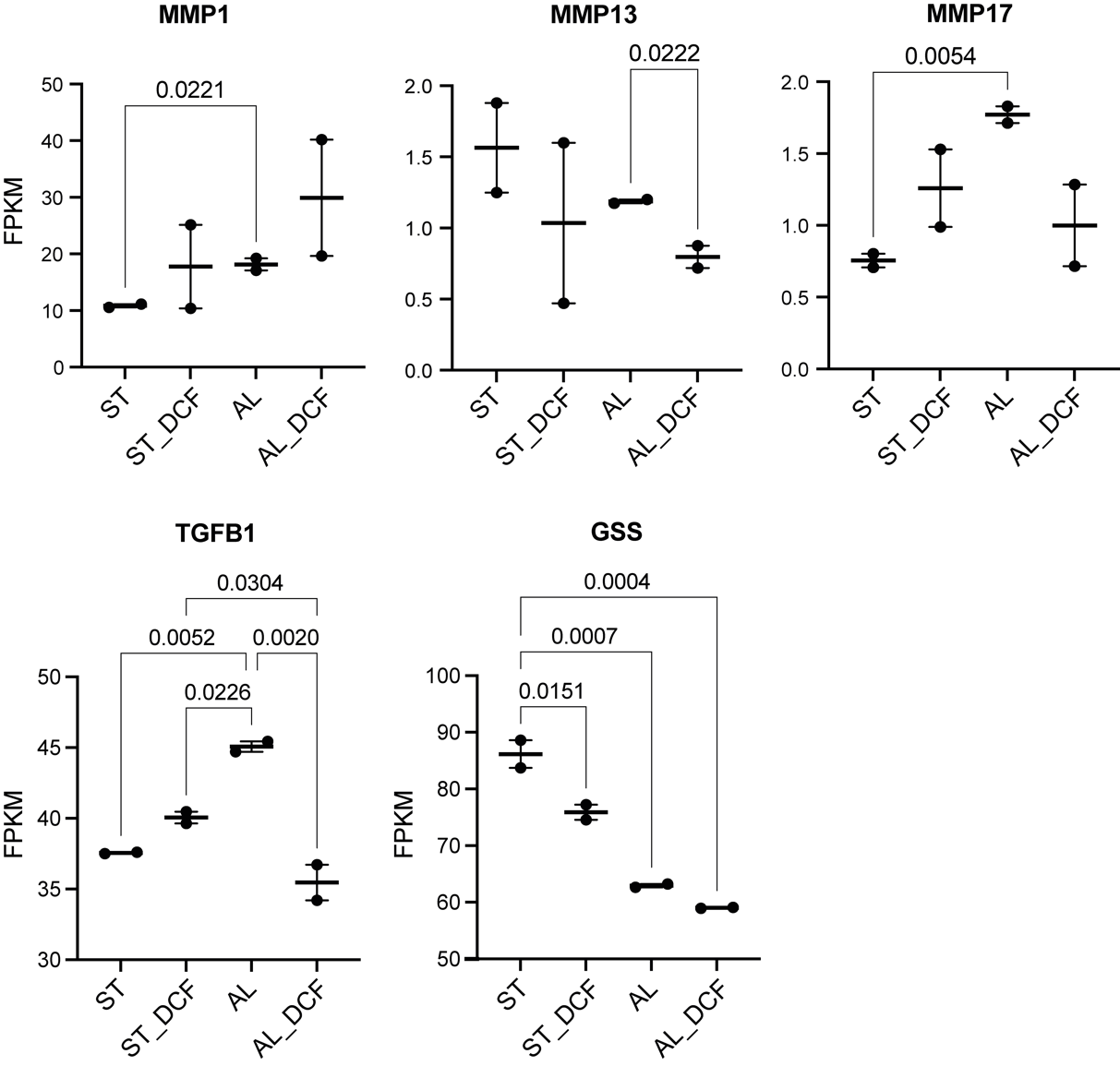


**Supplementary Figure 6.** The comparative gene expression (normalized FPKM values) in dot plot in which the mean of each group is indicated with a black line (data are presented in duplicates as means ± S.E.M). The *p*-values were determined using the Tukey HSD test and t-test comparison test.


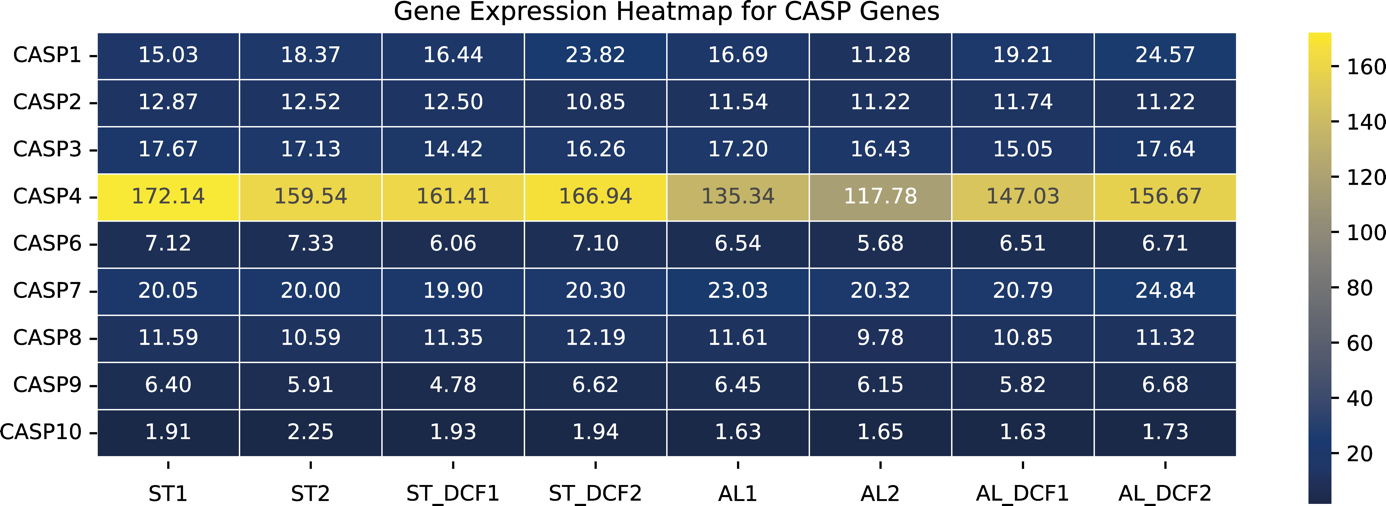


**Supplementary Figure 7.** Heatmap of apoptosis-related genes. Colored code bar indicates the gene expression (normalized FPKM values).


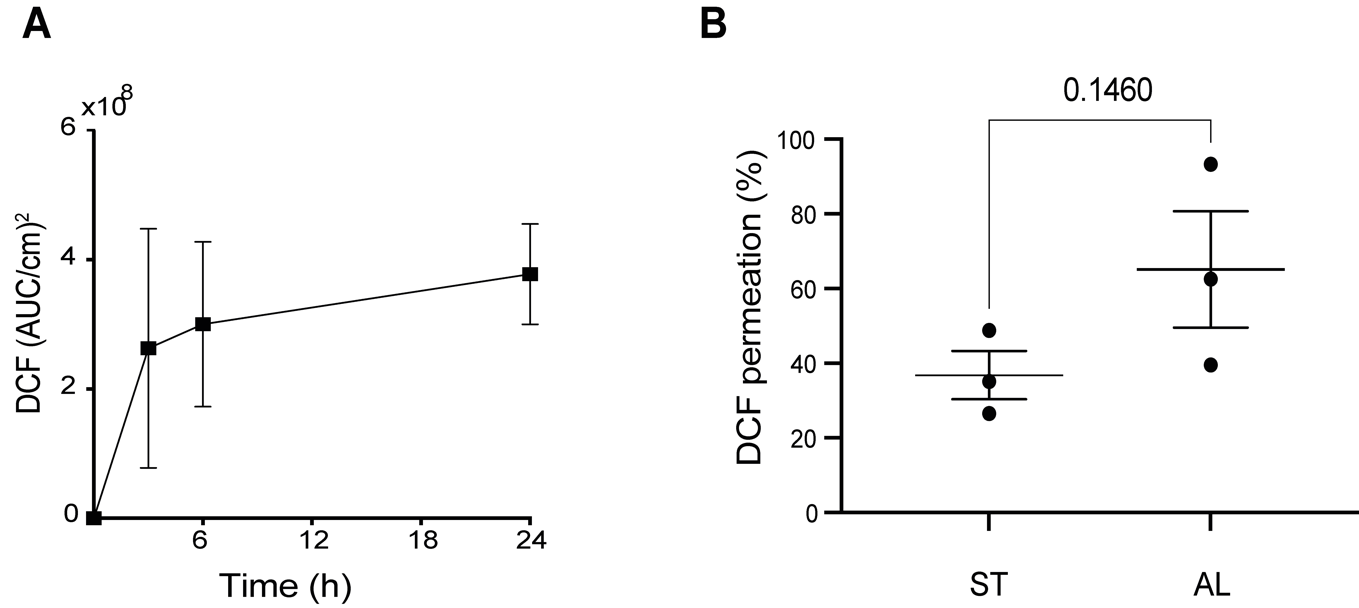


**Supplementary Figure 8. Diclofenac (DCF) accumulation in the basolateral compartment.** (A) DCF accumulation under AL stimulus. **(**B) DCF permeation percentage into the basolateral compartment after 3 h under ST and AL stimulus conditions**.** Data are presented in triplicates as means ± S.E.M. The *p*-values were determined using the t-test comparison test.

Supplementary Table 1. List of reagents and resources

| **Item** | **Maker** | **Catalog number** | **Details** |
| --- | --- | --- | --- |
| ZO-1antibody, Rabbit | Thermo Fisher | 61-7300 | IF 1:50 |
| P-gp antibody, Rabbit | Abcam | ab129450 | IF 1:50 |
| CK12 antibody, Mouse | Santacruz | sc-515882 | IF 1:200 |
| Phalloidin-iFluor 594 | Abcam | ab176757 |  |
| Diclofenac sodium | Wako | 043-22851 |  |
